# Effects of Thai Medicinal Herb Extracts with Anti-Psoriatic Activity on the Expression on NF-κB Signaling Biomarkers in HaCaT Keratinocytes 

**DOI:** 10.3390/molecules16053908

**Published:** 2011-05-10

**Authors:** Chanachai Saelee, Visa Thongrakard, Tewin Tencomnao

**Affiliations:** 1 Graduate Program in Clinical Biochemistry and Molecular Medicine, Department of Clinical Chemistry, Faculty of Allied Health Sciences, Chulalongkorn University, Bangkok, Thailand; 2 Center for Excellence in Omics-Nano Medical Technology Development Project, Department of Clinical Chemistry, Faculty of Allied Health Sciences, Chulalongkorn University, Bangkok, Thailand

**Keywords:** Thai medicinal herbs, NF-κB signaling biomarkers, psoriasis, keratinocytes, gene expression

## Abstract

Psoriasis is a chronic inflammatory skin disorder characterized by rapid proliferation of keratinocytes and incomplete keratinization. Discovery of safer and more effective anti-psoriatic drugs remains an area of active research at the present time. Using a HaCaT keratinocyte cell line as an *in vitro* model, we had previously found that ethanolic extracts from three Thai medicinal herbs, namely *Alpinia galanga*, *Curcuma longa* and *Annona squamosa*, possessed anti-psoriatic activity. In the current study, we aimed at investigating if these Thai medicinal herb extracts played a molecular role in suppressing psoriasis via regulation of NF-κB signaling biomarkers. Using semi-quantitative RT-PCR and report gene assays, we analyzed the effects of these potential herbal extracts on 10 different genes of the NF-κB signaling network in HaCaT cells. In accordance with our hypothesis, we found that the extract derived from *Alpinia galanga* significantly increased the expression of TNFAIP3 and significantly reduced the expression of CSF-1 and NF-κB2. *Curcuma longa* extract significantly decreased the expression of CSF-1, IL-8, NF-κB2, NF-κB1 and RelA, while *Annona squamosa* extract significantly lowered the expression of CD40 and NF-κB1. Therefore, this *in vitro* study suggested that these herbal extracts capable of functioning against psoriasis, might exert their activity by controlling the expression of NF-κB signaling biomarkers.

## 1. Introduction

Psoriasis, a chronic inflammatory skin disease affecting about 2–3% of the worldwide population, is characterized by hyperproliferation and abnormal differentiation of keratinocytes . At present, psoriasis is incurable, and its cause remains unelucidated. However, many studies have reported various factors contributing to the pathogenesis of psoriasis, including genetics, the immune system and environmental conditions, thus recognizing it as a multifactorial disease . Recently, a genome-wide scan study in 1,409 psoriasis cases and 1,436 controls revealed associations between three genes of the IL-23 pathway (IL23A, IL23R and IL12B), two genes of nuclear factor-κB (NF-κB) pathway (TNIP1 and TNFAIP3) and psoriasis [5].

NF-κB transcription factors play a critical role in the regulation of several genes, thus governing many biological effects including apoptosis, immune response and inflammatory processes . Therefore, dysfunction of NF-κB molecular network has been shown to contribute to inflammatory diseases such as psoriasis. The mammalian NF-κB family consists of five members, which are RELA (p65), NF-κB1 (p50; p105), NF-κB2 (p52; p100), c-Rel and RelB . Both mRNA and protein levels of p50, p65, RelB and c-Rel have been found to be increased in cytoplasm of primary cultured keratinocytes and keratinocyte cell line, thus highlighting a molecular involvement of NF-κB in keratinocyte growth [9]. A low level of p65 in normal skin cells has been reported, while a high expression in psoriatic lesions was demonstrated using immunohistochemistry [10]. Furthermore, imbalance between pro-apoptotic and anti-apoptotic activity of NF-κB proteins has been demonstrated to cause differentiation and hyperproliferation in psoriatic lesions, rather than in normal cells [11]. In animal models, IκB-α knockout mice were found to have hyperplasia of epidermis and infiltration of white blood cells in dermis due to highly unregulated function of p65 [12]. To date, treatment of psoriasis has been reported to result not only in side effects and drug resistance [13], but also economic impact [14] and quality of life [15] issues. Thus, an alternative treatment by herbal extract might provide an effective, safe and inexpensive therapy. In our previous *in vitro* screening of 11 Thai medicinal herbs used for treating skin diseases, we identified three herbal extracts, *Alpinia galanga* (rhizomes), *Curcuma longa* (rhizomes) and *Annona squamosa* (leaf), showing anti-psoriatic activity against a HaCaT cell line chosen as an *in vitro* model [16]. In particular, our study also demonstrated that an ethanolic extract from *Alpinia galanga* (rhizomes) resulted in a decrease in both mRNA and transforming growth factor-alpha (TGF-α) protein, a biomarker gene increased in psoriatic lesions [17].

However, little is known about related mechanisms of these Thai herbal plants, particularly their molecular roles in suppressing psoriatic activity. The effect of Thai medicinal herb extracts may have other mechanisms in anti-psoriasis such as inhibition of NF-κB transcription factors and their downstream targets.

The objective of this study was thus to investigate the molecular involvement of NF-κB signaling network biomarkers associated with psoriasis after treatment with Thai medicinal herb extracts, using cultured HaCaT cells as an *in vitro* model. Expression of NF-κB signaling network biomarkers was detected by semi-quantitative reverse transcriptase-polymerase chain reaction (RT-PCR) and reporter gene assay. We expected to give insights into an understanding of effects of these Thai medicinal herb extracts on the expression of NF-κB biomarkers. 

## 2. Results and Discussion

### 2.1. Effect of Thai Herbal Extracts on NF-κB Signaling Network Biomarkers Expression in HaCaT Cells

**Figure 1:**
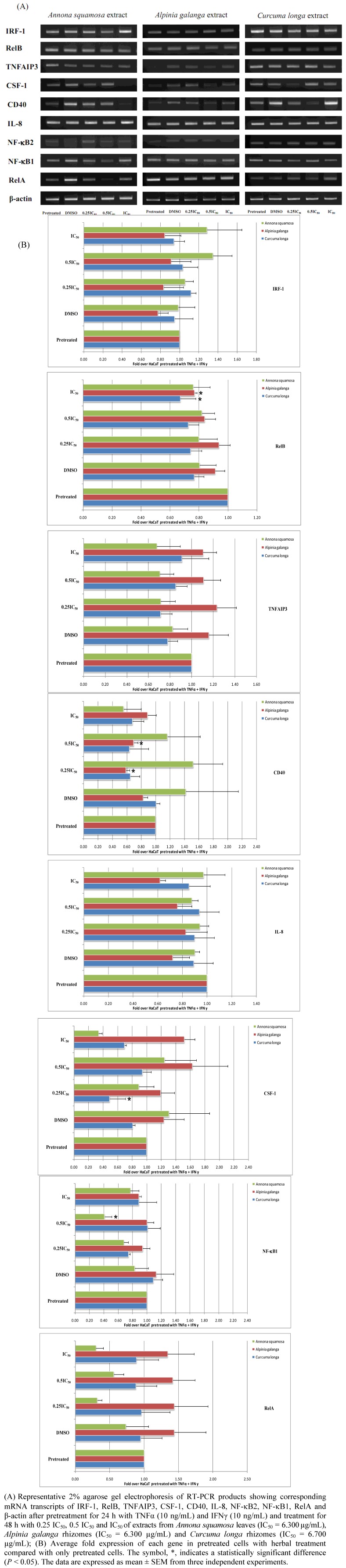
The effect of Thai medicinal herb extracts on the expression of mRNA transcripts of NF-κB signaling network biomarkers in HaCaT cells pretreated for 24 h with both proinflammatory cytokines (TNFα and IFNγ) and treated for 48 h with increasing concentrations of each herbal extract type.

Semi-quantitative RT-PCR was employed for evaluating the effect of each crude ethanolic extractof Thai medicinal plant spossessing anti-psoriatic property on the mRNA expression of NF-κB signaling network biomarkers. Results based on our initial experimental condition, pretreating cells with TNFα/IFNγ for 24 h and treating cells with each herbal extract at different concentrations for 48 h are shown in Figure 1. 

We found that the ethanolic extract of *Annona squamosa* at 3.150 µg/mL (0.5 IC_50_) significantly decreased NF-κB1 mRNA transcripts (*P* < 0.05). *Alpinia galanga* extract at 6.300 µg/mL (IC_50_) significantly decreased RelB mRNA transcripts (*P* < 0.05). This extract at 3.150, and 1.575 µg/mL (0.5 IC_50_ and 0.25 IC_50_) also significantly decreased CD40 mRNA transcripts (*P* < 0.05). It also significantly decreased CD40 mRNA transcripts when cells were treated at 3.150 µg/mL (0.5 IC_50_) (*P* < 0.05). *Curcuma longa* extract significantly reduced RelB and CSF-1 mRNA transcripts when cells were treated at 6.700 µg/mL (IC_50_) and 1.675 µg/mL (0.25 IC_50_), respectively (*P* < 0.05). 

**Figure 2:**
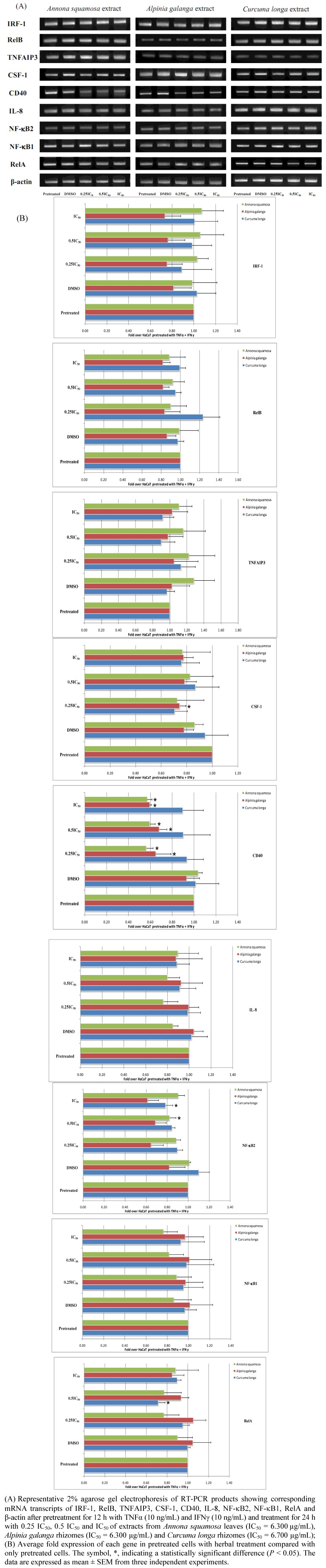
The effect of Thai medicinal herb extracts on the expression of mRNA transcripts of NF-κB signaling network biomarkers in HaCaT cells pretreated for 12 h with both proinflammatory cytokines and treated for 24 h with increasing concentrations of each herbal extract type.

Subsequent assays for the corresponding reporter genes with regard to the NF-κB signaling network biomarkers carried out after pretreatment with TNFα and IFNγ for 24 h. were not successful due to massive cytotoxicity. Therefore, we modified our experimental conditions by pretreating HaCaT cells for only 12 h, and the expression of NF-κB signaling network biomarkers was determined by RT-PCR after treatment for 24 h using each herbal extract (Figure 2). *Annona squamosa* extract at concentrations of 1.575, 3.150 and 6.300 µg/mL, corresponding to 0.25 IC_50_, 0.5 IC_50_ and IC_50_, respectively, significantly diminished CD40 mRNA transcripts (*P* < 0.05), and this particular extract only at 3.150 µg/mL (0.5 IC_50_) significantly reduced NF-κB2 mRNA transcripts (*P* < 0.05). *Alpinia galanga* extract at 6.300, 3.150, and 1.575 µg/mL (IC_50_, 0.5 IC_50_ and 0.25 IC_50_) significantly decreased CD40 mRNA transcripts (*P* < 0.05). This extract at 1.575 µg/mL (0.25 IC_50_) also significantly decreased CSF-1 mRNA transcripts (*P* < 0.05). With respect to *Curcuma longa* extract, it significantly suppressed NF-κB2 and RelA mRNA transcripts when cells were treated at 6.700 µg/mL (IC_50_) and 3.350 µg/mL (0.5 IC_50_), respectively (*P* < 0.05).

**Figure 3:**
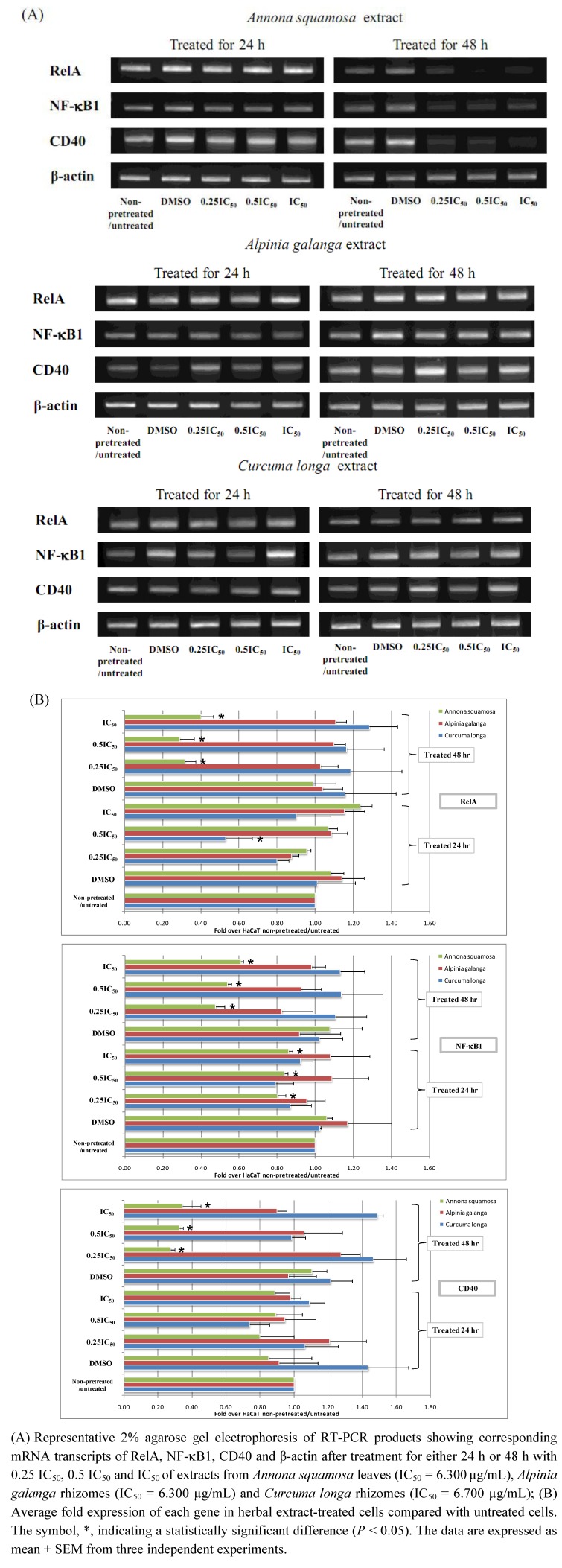
The effect of Thai medicinal herb extracts on the expression of mRNA transcripts of NF-κB signaling network biomarkers in proinflammatory cytokines-non-pretreated HaCaT cells treated with increasing concentrations of each herbal extract type for either 24 h or 48 h.

However, the expression of VCAM1, RelA, NF-κB1 and CD40 promoter constructs was not successfully carried out using the reporter gene assay under these conditions, as they generated extremely low luciferase signals for both firefly and *Renilla* ones. Therefore, we modified the experimental conditions for these four genes of interest, treating cells with each Thai herbal extract for 24 h and 48 h, without using proinflammatory cytokines for initial pretreatment step. As shown in Figure 3, NF-κB1 mRNA transcripts were significantly decreased when HaCaT cells were treated with *Annona squamosa* extract at 1.575, 3.150, and 6.300 µg/mL for both 24 h and 48 h (*P* < 0.05), while RelA and CD40 mRNA transcripts were significantly downregulated by this herbal extract at all concentrations applied when treating cells for the period of 48 h only (*P* < 0.05). For *Curcuma longa* extract, it significantly decreased RelA mRNA transcripts when treating HaCaT cells for 24 h at concentration of 3.350 µg/mL (0.5 IC_50_) (*P* < 0.05). VCAM1 mRNA transcripts were not found to be expressed in HaCaT cells regardless of conditions used in the present study.

### 2.2. Effect of Thai Medicinal Herb Extracts on Functional Promoters of NF-κB Signaling Network Biomarkers in HaCaT Cells

We used the reporter gene approach for assaying functional promoters of numerous genes in the NF-κB signaling network. In particular, reporter genes were analyzed in HaCaT cells after 12 h of pretreatment with proinflammatory cytokines and 24 h of treatment with each herbal extract in order to correlate with the RT-PCR approach with regard to using the similar time course. 

**Figure 4:**
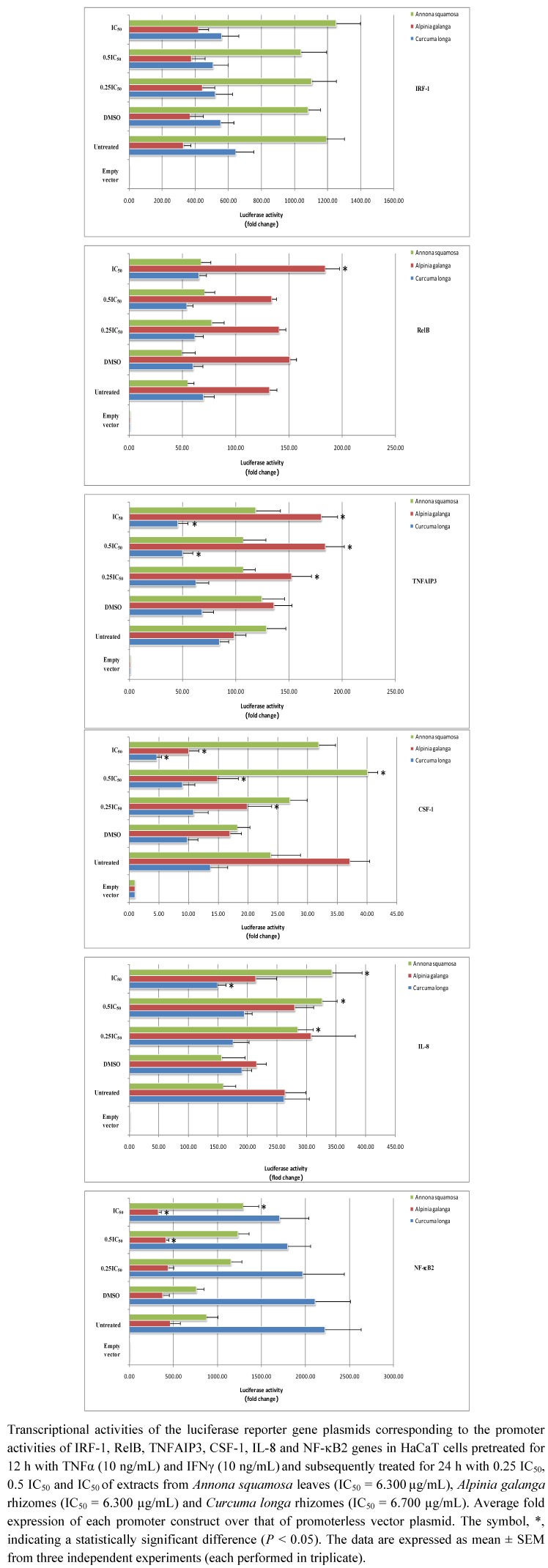
The effect of Thai medicinal herb extracts on promoter activity of NF-κB signaling network genes in HaCaT cells pretreated for 12 h with both proinflammatory cytokines and treated for 24 h with increasing concentrations of each herbal extract type.

In Figure 4, the reporter gene assay result shows the effect that *Annona squamosa* extract significantly increased IL-8 promoter activity when using 1.575 µg/mL (0.25 IC_50_) for treating cells (*P* < 0.05), whereas it significantly enhanced CSF-1 and IL-8 promoter activity at 3.150 µg/mL (0.5 IC_50_) (*P* < 0.05). In addition, it significantly promoted IL-8 and NF-κB2 promoter activity at 6.300 µg/mL (IC_50_) (*P* < 0.05). As for *Alpinia galanga*, its extracts at 1.575 µg/mL (0.25 IC_50_) and 3.150 µg/mL (0.5 IC_50_) significantly decreased CSF1 and NF-κB2 promoter activity (*P* < 0.05), but significantly increased TNFAIP3 promoter activity (*P* < 0.05). Also, at a concentration of 6.700 µg/mL (IC_50_), it significantly enhanced RelB and TNFAIP3 promoter activity (*P* < 0.05). As far as *Curcuma longa* was concerned, its extract at 3.350 µg/mL (0.5 IC_50_) significantly decreased TNFAIP3 promoter activity (*P* < 0.05), while significantly decreasing TNFAIP3, CSF-1 and IL-8 promoter activity at 6.700 µg/mL (IC_50_) (*P* < 0.05). 

**Figure 5:**
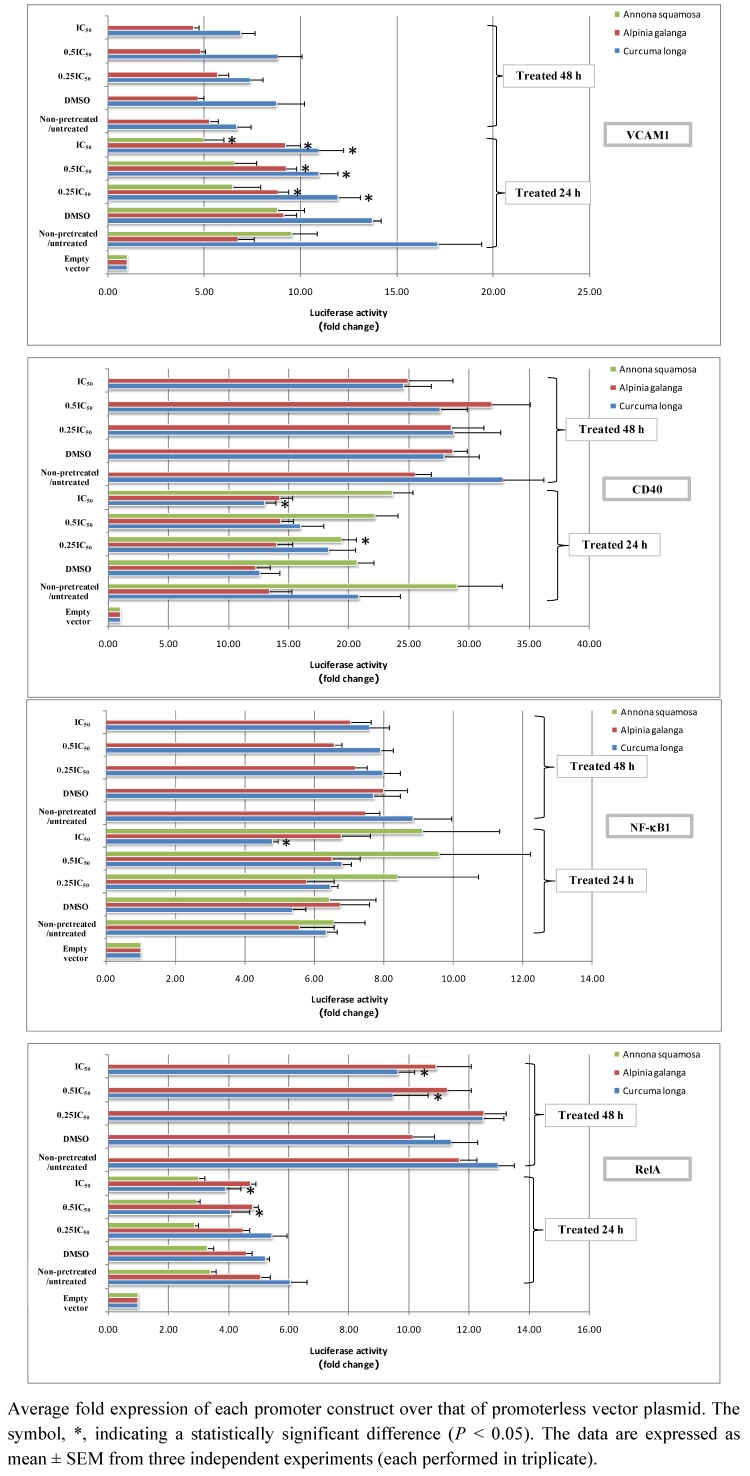
The effect of Thai medicinal herb extracts on promoter activity of NF-κB signaling network genes in proinflammatory cytokines-non-pretreated HaCaT cells treated with increasing concentrations of each herbal extract type for either 24 h or 48 h. Transcriptional activities of the luciferase reporter gene plasmids corresponding to the promoter activities of VCAM-1, CD40, NF-κB1 and RelA genes in HaCaT cells treated for either 24 h or 48 h with 0.25 IC_50_, 0.5 IC_50_ and IC_50_of extracts from *Annona squamosa* leaves (IC_50_ = 6.300µg/mL), *Alpinia galanga* rhizomes(IC_50_ = 6.300 µg/mL)and *Curcuma longa* rhizomes (IC_50_ = 6.700 µg/mL).

Since expression of VCAM1, CD40, NF-κB1 and RelA reporter promoters could not be successfully detected under these conditions, we altered the experimental protocol by assaying reporter genes after treatment with each Thai herbal extract for 24 h and 48 h in the absence of a pretreatment step with the proinflammatory cytokines. As shown in Figure 5, after treatment for 24 h, *Annona squamosa* extract significantly depleted CD40 and VCAM1 promoter activity when treating cells at 1.575 µg/mL (0.25 IC_50_) and 6.300 µg/mL (IC_50_), respectively (*P* < 0.05). 

After 48 h of *Annona squamosa* treatment, neither firefly nor *Renilla* luciferase signals could be found the assays for four reporter genes were performed. This might be due to the severe cellular damage caused by our observation since we noticed HaCaT cells displayed morphological changes, unhealthy appearance and unclear cell boundaries. For *Alpinia galanga* extract, it significantly increased VCAM1 promoter activity after 24 h treatment at all concentrations applied (*P* < 0.05). With regard to treatment using *Curcuma longa* extract for 24 h, it significantly reduced VCAM1 promoter activity at all concentrations, while significantly decreasing RelA promoter activity only at the two higher concentrations, 3.350 µg/mL (0.5 IC_50_) and 6.700 µg/mL (IC_50_) (*P* < 0.05). In addition, this particular extract at 6.700 µg/mL (IC_50_) significantly reduced CD40 and NF-κB1 promoter activity (*P* < 0.05). In accordance with treatment of 24 h, RelA promoter activity was also significantly promoted when treating cells with the two higher concentrations of this plant extract for 48 h (*P* < 0.05).

The NF-κB signaling network has an important role in modulating the number and function of skin cells since it has been shown to control the apoptotic process in skin and involved in the inflammatory process . Thus, an imbalance of NF-κB function leads to skin diseases resulting from hyper-proliferation and inflammation such as psoriasis. Recently, the study of Nair *et al.* [5] revealed a highlighted molecular involvement of NF-κB in psoriasis. Thus, we were so interested in investigating the *in vitro* effects of Thai medicinal herb extracts, which have been reported to possess anti-psoriatic activity [16]. We chose the HaCaT cell line for this study because it has been shown to be a suitable *in vitro* model and has been used in numerous studies [18,19,20]. 

In analyzing mRNA expression and promoter activity of genes in NF-κB network, which consisted of CD40, CSF-1, IL-8, IRF-1, NF-κB2, NF-κB1, RelA, RelB, TNFAIP3 and VCAM1, we found that mRNA expression could be detected by stimulating cells by TNFα and IFNγ for 24 h and treating cells by Thai herbal extract for 48 h, but we could not detected the expression of promoter activity due to cytotoxicity. Thus, we changed the period of cell stimulation by proinflammatory cytokines from 24 h to 12 h and reduced the period of Thai herbal extract treatment from 48 h to 24 h. However, expression of promoter activity of VCAM1, CD40, NF-κB1 and RelA could not be detected if transfection using one of these four reporter constructs into HaCaT cells in the subsequent addition of proinflammatory cytokines. Still, we could not provide any explanation for this unexpected finding. To solve the problem of cell damage and the unknown phenomenon from proinflammatory cytokines, we avoided pretreatment using proinflammatory cytokines, but performed herbal treatment for 24 h and 48 h. 

In the present study, we found that Alpinia galanga extract could reduce the expression of NF-κB signaling biomarkers via an increased expression of TNFAIP3, the molecule inhibiting NF-κB [21]. Therefore, this extract evidently decreased the expression of CSF-1 and NF-κB2 gene. This particular extract decreased the mRNA expression of CD40, IL8 and RelB, but this finding was not supported by the reporter gene assay. For Curcuma longa extract, it decreased such transcription factors as NF-κB1, NF-κB2 and RelA. Thus, we also found the reduced expression of CSF-1 and IL-8 due to the effect of this particular extract. Annona squamosa extract decreased the expression of CD40, known as a stimulator of the NF-κB1 classical pathway [22]. This herbal extract was also found to decrease mRNA of IL-8, NF-κB2, RelA and RelB genes in this study although this result was not in agreement with the result from the reporter gene assay. In psoriasis, NF-κB has been reported to be expressed at levels higher in lesional skin biopsies than those in normal cells based on immunohistochemistry [23]. The same is true for the expression of IL-8 [24], CD40 [25] and VCAM1 [26] since there have been increased in lesional skin. Although eight of ten genes were found to be expressed in either HaCaT cells or skin biopsy specimens, two genes, CSF-1 and VCAM1, have never been reported to be expressed in HaCaT cells. For the first time, we detected the mRNA expression of CSF-1 gene in this study. However, we could not find VCAM1 expression in the present work as it might be only specifically expressed in other cells rather than keratinocytes. Certain conflicting results between mRNA detection and reporter gene assay in our current study might be due to the fact that the promoter length include was only limited to about 1 kb. 

With regard to these Thai herbal extracts displaying anti-psoriatic activity, we found their effects on certain NF-κB biomarkers for each extract. Therefore, it should be noted that the identity of the active compound(s) in these herbal extracts should be addressed. Interestingly, curcumin in Curcuma longa and quercetin in Annona squamosa have been revealed to possess an anti-inflammatory effect [27,28], while 1’-acetoxychavicol acetate in Alpinia galanga has been reported to inhibit NF-κB [29]. In the future, discoveries of active constituents in these Thai herbal extracts affecting mRNA and NF-κB signaling network biomarkers should be carried out. Combination of these herbal compounds derived from various plants may also be needed in order to target the molecules of NF-κB signaling network for not only safer, but better in treatment of psoriasis. 

## 3. Experimental

### 3.1. Plant Materials and Preparation of Herbal Extracts [16]

In brief, the Thai traditional medicinal plants, *Curcuma longa*, *Alpinia galanga* and *Annona squamosa*, were collected from the Princess Maha Chakri Sirindhorn Herbal Garden (Rayong Province, Thailand). They were authenticated by Professor Dr. Thaweesakdi Boonkerd (Department of Botany, Faculty of Science, Chulalongkorn University, Thailand). The respective voucher specimens [013396 (BCU), 013397 (BCU) and 013399 (BCU)] were deposited at the Professor Kasin Suvatabhandhu Herbarium, Department of Botany, Faculty of Science, Chulalongkorn University, Thailand. All plants were extracted by maceration at room temperature with ethanol (Merck, Hohenbrunn, Germany) using a 1:5 (w/v) ratio in a shaking incubator at 120 rpm for 48 h. The medicinal plant extracts were filtered, and the residues were subsequently extracted twice more. After the two filtrates were combined, the crude extracts were concentrated using the MiVac Quattro concentrator at 45 °C. Finally, the resulting crude extracts were dissolved in dimethyl sulphoxide (DMSO, Merck) as stock solutions (100 mg/mL), stored at −20 °C and protected from light. Prior to incubation with HaCaT cells, the crude extracts were filtered through a 0.2 µm pore size filter (Corning Inc., Corning, NY, USA).

### 3.2. Cell Culture

HaCaT cells (Cell line service, Heidelberg, Germany), the immortalized human epidermal keratinocyte cell line, were cultured in Dulbecco’s Modified Eagle Medium/High glucose (DMEM/HG) (Hyclone, Logan, UT, USA) with 10% fetal bovine serum, 100 U/mL penicillin and 100 µg/mL streptomycin (Hyclone). The cells were cultured at 37 °C in a humidified atmosphere at 5% CO_2_ for 24 h. 

### 3.3. Treatment of HaCaT Cells Using Thai Medicinal Herb Extracts

Plant materials and anti-psoriatic activity assays were previously described by our group, and IC_50 _value for each plant used was also addressed, particularly those Thai medicinal herbs having anti-psoriatic effect, *Annona squamosa, Alpinia galanga*, and *Curcuma longa* [16]. HaCaT cells were seeded in six-well plates at cell densities of 1 × 10^6^ cells. The cells were pretreated with pro-inflammatory cytokines, TNFα (10 ng/mL) and IFNγ (10 ng/mL) (Peprotech, Rocky Hill, NJ, USA), for 12 h and 24 h or were left nonpretreated. After that, pretreated cells were washed with Phosphate Buffer Saline (PBS, Hyclone) and treated with various concentrations of each plant extract: 6.300, 3.150, 1.575 µg/mL (IC_50_ = 6.300) of *Alpinia galanga* rhizome extract, *Annona squamosa* leaf extract or 6.700, 3.350, 1.675 µg/mL (IC_50_ = 6.700) of *Curcuma longa* rhizome extract for the period of either 24 h or 48 h at 37 °C in a humidified atmosphere at 5% CO_2_. Control cells were either only pretreated cells or left nonpretreated cells depending on the experimental design. 

### 3.4. Semi-quantitative Reverse Transcriptase-polymerase Chain Reaction (RT-PCR)

Treated cells were washed with PBS. Total RNA was isolated from HaCaT cells by Trizol^®^ reagent (Invitrogen, Carlsbad, CA, USA) according to the manufacturer’s instructions. After extraction, total RNA was measured at 260 nm for quantification. Before performing the RT reaction, total RNA was adjusted to the final concentration of 1 μg/uL, and RNA was treated with 10^−5^ U deoxyribonuclease I (DNase I) (Invitrogen) for 30 min at 37 °C. DNase I-treated RNA was reverse transcribed by ImProm-II^TM ^Reverse Transcription System (Promega, Madison, WI, USA) with oligo(dT)_17_ primer following the manufacturer’s protocol. For amplification of the cDNA, each desired DNA fragment was amplified for 35–40 cycles using each gene-specific primer pair as listed in Table 1. 

**Table 1:** Specific nucleotide primer used in RT-PCR.

Name	Sequences		PCR product lengths	Ref.
β-actin	forward	5’ ACG GGT CAC CCA CAC TGT GC 3’	656 bp	[30]
	reverse	5’ CTA GAA GCA TTT GCG GTG GAC GAT 3’		
IRF-1	forward	5’ AAC AAG GGC AGC TCA GCT GT 3’	450 bp	[31]
	reverse	5’ TGT TGG CTG CCA CTC CGA CT 3’		
VCAM-1	forward	5’ AGT CAG GAA TTT CTG GAG GAT GC 3’	229 bp	[32]
	reverse	5’ GCA GCT TTG TGG ATG GAT TCA 3’		
RelB	forward	5’ TCC CAA CCA GGA TGT CTA GC 3’	160 bp	[33]
	reverse	5’ AGC CAT GTC CCT TTT CCT CT 3’		
TNFAIP3	forward	5’ TTC AAG CAG ATG TAT GGC TAA CC 3’	267 bp	[34]
	reverse	5’ CCT TGG GCT GAA TCT GAC AT 3’		
CSF-1	forward	5’ ATG ACA GAC AGG TGG AAC TGC CAG 3’	438 bp	[35]
	reverse	5’ TCA CAC AAC TTC AGT AGG TTC AGG 3’		
CD40	forward	5’ AGA GTT CAC TGA AAC GGA ATG CC 3’	461 bp	[36]
	reverse	5’ ACA GGA TCC CGA AGA TGA TGG 3’		
IL-8	forward	5’ CTG CGC CAA CAC AGA AAT TA 3’	238 bp	[37]
	reverse	5’ ATT GCA TCT GGC AAC CCT AC 3’		
NF-κB2	forward	5’ CAG TGA GAA GGG CCG AAA GAC 3’	421 bp	This study
	reverse	5’ CAG GGG CAG GGA GAA GGA G 3’		
NF-κB1	forward	5’ AGC CCC CAA TGC ATC CAA CTT 3’	402 bp	[7]
	reverse	5’ CAA CCG CCG AAA CTA TCC GAA AAA 3’		
RelA	forward	5’ AGC GCA TCC AGA CCA ACA ACA ACC 3’	419 bp	[7]
	reverse	5’ CCG CCG CAG CTG CAT GGA GAC AC 3’		

PCR reaction conditions consisted of 1 min denaturation at 94 °C, a 1 min annealing at 55 °C for TNFAIP3, IRF-1, RelB, IL-8 and NF-κB1, 60 °C for β-actin, 58.4 °C for NF-κB2, 55.9 °C for CD40, 58 °C for CSF1 and 67.7 °C for RelA, and a 1 min extension at 72 °C. The products of RT-PCR were performed on 2% agarose gel electrophoresis and visualized by ethidium bromide staining. All band densities were analyzed by the GeneTools program in a G:Box (Syngene, Cambridge, UK).

### 3.5. Plasmids

The promoterless luciferase expression vector (pSGG_prom vector) and NF-κB Biomarker Set consisting of eight luciferase reporter promoter gene constructs -CD40 (1,081 bp), CSF-1 (914 bp), IL-8 (1,085 bp), IRF-1 (958 bp), NF-κB2 (1,496 bp), RelB (1,062 bp), TNFAIP3 (1,039 bp), VCAM1 (1,061 bp)- were kindly provided by SwitchGear Genomics (Menlo Park, CA, USA). The pRL-CMV (*Renilla* luciferase) internal control plasmid (a generous gift from Dr. Robert K. Yu, Institute of Molecular Medicine and Genetics, Georgia Health Sciences University, USA) was used in this study.

### 3.6. Construction of Promoter Reporter Plasmids

In the present study, we made two additional reporter gene constructs (NF-κB1 and RelA) since they were also members of NF-κB family. Genomic DNA was extracted from HaCaT cells in order to use as a PCR template for amplifying promoters of both genes. For generating the promoter fragment, PCR was performed using specific primers encompassing position −979 to +15 (forward primer containing *Mlu*I restriction site, 5-cttgACGCGTGCTAAGCTTTCAGTTGT-3 and reverse primer containing *Bgl*II restriction site, 5-ccAGATCTCGCTCACTCTCTCACTTCCT-3) for NF-κB1 and specific primers encompassing position −1061 to +165 (forward primer containing *Nhe*I restriction site, 5-acGCTAGCCGTTAGGAGCCTTCTCAC-3 and reverse primer containing *Hind*III restriction site, 5-cacAAGCTTTCCCTCTTCTCAAGTGCC-3) for RelA. PCR conditions consisted of 30 s denaturation at 94 °C, a 30 min annealing at 58.1 °C for NF-κB1 promoter and 60.4 °C for RelA promoter, 58.4 °C for NF-κB2, and a 2 min extension at 72 °C. The PCR products were purified and cloned into pGEM-T Easy vector (Promega). Subsequently, target DNA fragments were digested from the clones with *Mlu*I/*Bgl*II and *Nhe*I/*Hind*III for NF-κB1 and RelA, respectively. Each corresponding promoter fragment was then purified and subcloned into restriction sites upstream of the luciferase gene of pSGG_prom empty vector. Finally, newly cloned reporter constructs were verified by DNA sequencing and used for reporter gene assays. 

### 3.7. Reporter Gene Assay

For transient transfection, HaCaT cells were cultured to reach about 70% confluence in six-well plates. Transfection of reporter plasmids into HaCaT cells was performed in DMEM without serum and antibiotics using FuGene HD reagent (Roche, Mannheim, Germany) following a procedure described by the manufacturer with little modifications. In particular, HaCaT cells were transfected with 2 µg of each reporter construct and cotransfected with 600 ng of pRL-CMV vector. Following 8 h of incubation with the transfection complex, the medium was changed to DMEM/HG with 10% fetal bovine serum, 100 U/mL penicillin and 100 µg/mL streptomycin, and cells were pretreated with proinflammatory cytokines, TNFα (10 ng/mL) and IFNγ (10 ng/mL) for 12 h and 24 h. Then, cells were treated with 6.300, 3.150, 1.575 µg/mL (IC_50 _= 6.300) of *Alpinia galanga* rhizome extract, *Annona squamosa* leaf extract, and 6.700, 3.350, 1.675 µg/mL (IC_50 _= 6.700) of *Curcuma longa* rhizome extract, for 24 h and 48 h or were left nonpretreated prior to lysis. The lysate was analyzed for the firefly and *Renilla* luciferase activities by Dual-Luciferase^®^ Reporter Assay System (Promega) following a procedure given by the manufacturer. VICTOR^3^ luminometer (PerkinElmer, Waltham, MA, USA) was used for measuring chemiluminescence. Luciferase signals were normalized to *Renilla* luciferase within each sample.

### 3.8. Statistical Analysis

Data are presented as the mean ± standard error of the mean (SEM) from three independent experiments for RT-PCR and nine independent experiments for reporter assays, and were analyzed by one-way analysis of variance (One-way ANOVA), followed by *pos-hoc* LSD test using pretreated cells or normal cells as a control group on the SPSS for Windows (version 17.0). Differences were considered to be significant at *P* < 0.05.

## 4. Conclusions

The present data of treatment with Thai medicinal herb extracts suggest that ethanolic extracts of *Alpinia galanga* could regulate NF-κB networks via decreased expression of CSF-1 and NF-κB2, while increased expression of TNFAIP3. *Curcuma longa* could decrease the expression of CSF-1, IL-8, NF-κB2, NF-κB1 and RelA. *Annona squamosa* could reduce the expression of CD40 and NF-κB1. Therefore, we demonstrated the effects of Thai medicinal herb extracts on downregulation of NF-κB signaling molecules, reflecting their potential use in treating such disease with inflammation and hyperproliferation as psoriasis. In the future, we hope that many research groups will attempt to study and develop Thai medicinal herbs for therapeutic applications with improved efficacy, safe and reduced cost. 
